# Down-regulation of progesterone receptor membrane component 1 (PGRMC1) in peripheral nucleated blood cells associated with premature ovarian failure (POF) and polycystic ovary syndrome (PCOS)

**DOI:** 10.1186/1477-7827-8-58

**Published:** 2010-06-10

**Authors:** Jens Schuster, Teresia Karlsson, Per-Olof Karlström, Inger Sundström Poromaa, Niklas Dahl

**Affiliations:** 1 Department of Genetics and Pathology, Uppsala University, 751 85 Uppsala, Sweden; 2 Department of Women's and Children's Health, Uppsala University, 751 85 Uppsala, Sweden; 3 Department of Clinical Science, Karolinska University Hospital, 141 86 Stockholm, Sweden

## Abstract

**Background:**

Progesterone receptor membrane component 1 (PGRMC1) is a member of a progesterone-binding complex implicated in female reproduction. We aimed i) to determine the natural expression of PGRMC1 in peripheral nucleated blood cells throughout the menstrual cycle and ii) to investigate any association between PGRMC1 levels in leukocytes and conditions characterized by reduced fertility.

**Methods:**

We analyzed PGRMC1 expression in peripheral leukocytes from 15 healthy cycling women over four weeks. Additionally, we determined PGRMC1 levels in samples from patients with premature ovarian failure (POF) and polycystic ovary syndrome (PCOS) as well as in healthy postmenopausal women and male controls. The levels of PGRMC1 protein in nucleated peripheral blood cells were quantified by Western blot analysis.

**Results:**

PGRMC1 levels did not vary significantly throughout the menstrual cycle. We observed a significant down-regulation of PGRMC1 in postmenopausal women and in patients with premature ovarian failure (POF) and polycystic ovary syndrome (PCOS) when compared to early follicular phase of healthy women.

**Conclusion:**

This study suggests that reduced levels of PGRMC1 in peripheral leukocytes are associated with perturbed ovulatory function.

## Background

An important mediator of progesterone's effects is its nuclear progesterone receptor (PGR). However, it has recently become evident that, at least in the uterus, many of the actions initialized by progesterone are mediated by non-genomic progesterone receptors [1], e.g. progesterone receptor membrane component 1 (PGRMC1; [2,3]). PGRMC1 was first described in 1998 [4] as a putative progesterone-binding membrane receptor of approximately 22 kDa [2]. The protein is expressed in a variety of tissues, e.g. liver, kidney, adrenal glands, uterus and leukocytes [2,5,6]. It was recently shown that PGRMC1 is part of a membrane complex that binds progesterone [2,3]. PGRMC1 is believed to be involved in progesterone signaling in the reproductive system and it mediates progesterone's anti-apoptotic effects on granulosa cells [2,5,7,8]. PGRMC1 binds to and positively regulates several members in the microsomal cytochrome P450 family of proteins, which are key players in intracellular sterol metabolism and steroidogenesis [5,6,9,10]. We have recently shown that reduced PGRMC1 levels are associated with premature ovarian failure (POF; [5]). Furthermore, a PGRMC1 missense variant identified in a patient with POF shows perturbed interaction with the P450 member CYP7A1 [5]. Recent studies have characterized the expression of PGRMC1 in uterine and placental tissues of murine origin [8,11]. Nothing is known about the natural variation of PGRMC1's expression throughout the human menstrual cycle or about the association of PGRMC1 levels to conditions with reduced fertility.

The aim of this study was to determine the natural expression levels of PGRMC1 in an easy accessible tissue throughout the menstrual cycle and to assess PGRMC1 levels in conditions associated with reduced fertility and anovulation. PGRMC1 is ubiquitously expressed and we selected nucleated peripheral blood cells for the analysis.

## Methods

### Healthy women and control groups

We enrolled 15 healthy cycling women (termed healthy females, HF) with a mean age of 27.5 ± 8.0 years, regular menses (28.0 ± 2.2 days, range 25 - 32 days) and without steroids or oral contraceptives during the past three months. Their control status was evaluated by medical history and a clinical examination. A retrospective assessment was used to establish their menstrual cycle pattern prior to inclusion, i.e. individuals were asked if they had regular menstrual cycles and their usual cycle length (in days) was noted. Venous blood samples were obtained twice weekly during four consecutive weeks. Estradiol and progesterone serum concentrations, the LH surge and records on the first day of menstrual bleeding were used to assign every sample to a distinct phase of a standardized 28-day menstrual cycle (table 1). Menstrual cycle phases were divided in early follicular phase (eFP; postmenstrual day 1-7), late follicular phase (lFP; postmenstrual day 8 - 11), preovulatory phase (PO; LH-1 - LH-2), early luteal phase (eLP; LH +1 - LH +5), mid-luteal phase (mLP; LH +6 - LH +10), late luteal phase (lLP; LH +11 - LH +14 or premenstrual days -1 - -4). If more than one sample from the same individual was assigned to the same cycle phase, mean values were calculated for the individual subject. Ovulation was determined in each subject by measuring levels of LH in urine samples (Clearplan, Unipath, Bedford, UK). Progesterone and estradiol were measured on Immulite 1000 (DPC, USA). For the estradiol assay the measured interval was 73-7300 pmol/l.

**Table 1:** Summary of samples from healthy women (HF) included in the study

**Menstrual Phase**^**a**^	**Number of samples**^**b**^	**Estrogen [pmol/l]**^**c**^	**Progesterone [nmol/l]**^**c**^
Early Follicular (eFP)	15	85.4 ± 29.7	1.02 ± 0.98
Late Follicular (lFP)	15	227.2 ± 195.1	0.64 ± 0.02
Preovulatory (PO)	7	780.2 ± 228.1	0.85 ± 0.42
Early Lutael (eLP)	13	211.3 ± 169.7	6.55 ± 4.50
Midluteal (mLP)	14	267.4 ± 101.3	27.36 ± 13.05
Late Luteal (lLP)	15	205.8 ± 193.7	10.30 ± 7.98

^a^Assigned phase integrating hormone measurements, LH test and information about menstrual bleeding

^b^Number of samples assigned to each distinctive phase

^c^Mean ± standard deviation

Five healthy natural postmenopausal women (PM; 62.0 ± 3.2 years of age) and four healthy men (HM) were included in the study as reference groups. The PM women were postmenopausal since 11.2 ± 3.0 years and were without any hormone replacement therapy during the past year. Mean FSH serum concentrations of PM were 62.0 ± 22.0 mIU/ml. Healthy men were between 30 and 75 years old (43.0 ± 18.1 years of age).

### PCOS and POF patients

Six women (mean age of 27.6 ± 6.9 years) with polycystic ovary syndrome (PCOS) and eight patients (mean age of 35.2 ± 6.3 years) with idiopathic premature ovarian failure (POF) were included. PCOS diagnosis was defined according to the Rotterdam criteria [12]. All three features had to be present for diagnosis 1) oligomenorrhea with eight or fewer menstruations in the previous 12 months or amenorrhea; 2) clinical and/or biochemical indications of hyperandrogenism; 3) polycystic ovaries on ultrasound examination (> 12 follicles 2 to 9 mm in diameter) and/or increased ovarian volume (> 10 ml). PCOS diagnosis also implied that no evidence of thyroid disease (normal s-TSH), adrenocortical dysfunction (normal 17-hydroxyprogesterone), or hyperprolactinemia (prolactin < 30 μg/ml) was present. All six PCOS patients had biochemical or clinical signs of hyperandrogenism. Mean free androgen index was 8.4 ± 5.4 and mean FSH level was 3.9 ± 1.4 mIU/ml. The POF group comprised three patients with primary amenorrhea and elevated FSH (>40 mIU/ml) and five patients with secondary amenorrhea (of which four had FSH values > 50 mIU/ml). Mean FSH serum concentration among POF patients was 49.8 ± 33.1 mIU/ml. PCOS and POF patients were sampled prior to any treatment of their symptoms.

### Ethical statement

The healthy women constitute a group of students at Uppsala University recruited by advertisement. Postmenopausal women, POF and PCOS patients were referred to the gynecologists (ISP and POK), Department of Obstetrics and Gynecology, Uppsala University Hospital. The healthy males are employed at Uppsala University and were interviewed and informed by JS prior to inclusion in the study. All participants are living in Sweden and gave a written informed consent prior to inclusion in the study. The study was approved by the Ethical Review board at Uppsala University, Sweden.

### Analysis of PGRMC1 expression

Total protein was isolated from EDTA blood samples as follows: Erythrocytes were lyzed by mixing blood samples with lysis buffer (115 mM NH_4_Cl, 10 mM KHCO_3_, 0.1 mM EDTA), incubating on ice for 15 minutes (repeated once) and subsequent collection of the remaining peripheral nucleated cells (PNBC) by centrifugation. Protein was extracted from the PNBC obtained using TRIZOL (Invitrogen) following manufacturer's protocols. Levels of PGRMC1 protein were determined by separating protein samples on a 12% SDS-PAGE (NuPage, Invitrogen) and subsequent transfer to PVDF Immobilon-FL membranes (Millipore) according to manufacturer's protocols. Proteins were detected using primary α-PGRMC1 (Sigma) and α-*β*-actin antibodies (Abcam), respectively. Detected proteins were visualized using IRD680- or IRD800 -labeled secondary antibodies, respectively (LiCor Biosciences). Western blots were analyzed using the Odyssey infrared imaging system, determining integrated intensities for each protein following the instructions manual (LiCor Bioscience). In parallel, *β*-actin was measured in the same samples and used for internal normalization (PGRMC1 level_lane N _= Integrated Intensity[PGRMC1]_lane N _/Integrated Intensity[*β*-actin]_lane N_). Each sample was analyzed two to four times (loading varying amounts) and the average of the independent measurements was used for further analysis to rule out experimental error.

### Statistical analysis

Data were analyzed using the programs Excel^® ^(Microsoft^®^) and Minitab Statistical Software™ v13.20 (Minitab INC). The differences between study groups were tested for significance as follows: First, data was tested for normal distribution (Anderson-Darling and Kolmogorov-Smirnov normality tests) and equal variance (Bartlett's and Levene's test). The data was not normally distributed and showed unequal variance. Differences within and between study groups were tested with the Kruskal-Wallis test and a significant *p*-value was obtained (*p *< 0.001). Subsequently, pairwise differences were investigated using a post hoc test (Mann-Whitney *U*-test).

## Results

**Figure 1:**
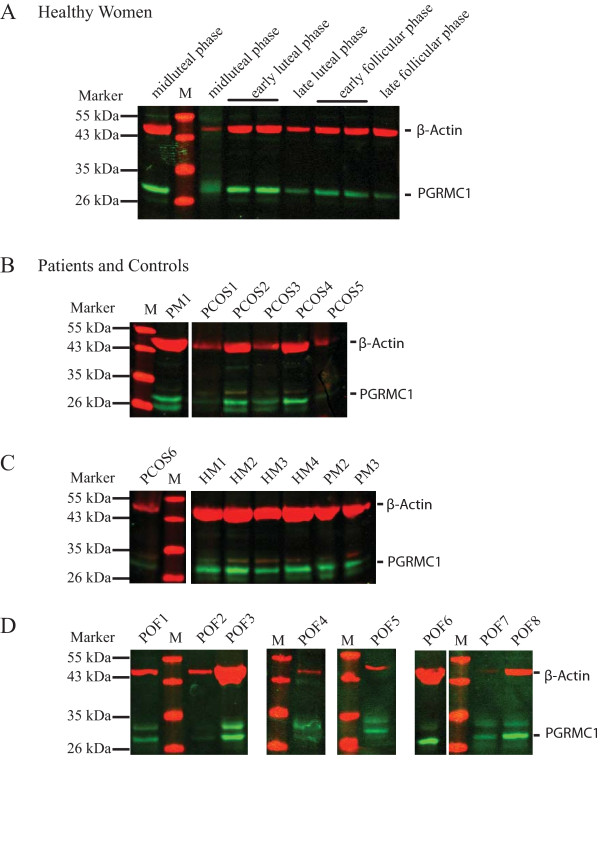
**Detection of PGRMC1 and β-actin by Western blot analysis in total protein preparations obtained from peripheral nucleated blood cells (PNBC)**. Representative western blot pictures illustrating detection in (A) healthy cycling women (HF; distinct phases are indicated above), (B-D) PCOS and POF patients and control groups (PM, natural menopausal women; HM, healthy men). Total protein preparations were separated on a 12% SDS-PAGE, transferred to a PVDF membrane and subsequently detected using α-PGRMC1 and α-β-actin antibodies. A protein standard was included and the band sizes are indicated to the left. Bands corresponding to PGRMC1 (green) and β-actin (red) are indicated.

**Figure 2:**
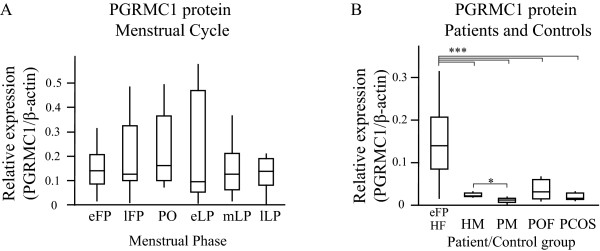
**Progesterone receptor membrane component 1 (PGRMC1) protein expression**. (A) Expression of PGRMC1 protein in healthy cycling women across an idealized 28-day menstrual cycle according to cycle phase and (B) in women with premature ovarian failure, polycystic ovary syndrome, and natural menopause. Western blots (example shown in figure 1) were analyzed using Odyssey infrared imaging system to determine integrated intensities for PGRMC1 and β-actin in the same sample. β-actin was used for normalization. Measured PGRMC1 levels were displayed as box plots (***p-value < 0.01; *p-value < 0.05; student's t-test). [*Menstrual cycle analysis *(HF: healthy women): eFP, lFP: early and late follicular phase; PO: preovulatory phase; eLP, mLP, lLP: early, mid- and late luteal phase; *Study groups*: HM: healthy men; PM: postmenopausal women; POF: premature ovarian failure; PCOS: polycystic ovary syndrome]

We obtained eight venous EDTA blood samples from each of 15 healthy women during four weeks. The time spans approximately one menstrual cycle and none of the women were on any hormonal treatment. Serum estrogen and progesterone levels were measured in each sample. Both hormones showed expected and normal profiles over the four weeks period (table 1). The hormone profile, LH surge and first day of menstrual bleeding were used to assign every sample to a distinct day of a standardized 28 day menstrual cycle (table 1). Subsequently, we analyzed PGRMC1 protein levels in total protein preparations derived from PNBCs by Western Blot analysis (figure 1). We established expression profiles for PGRMC1 throughout the menstrual cycle (figure 2A). The levels of PGRMC1 were found stable across the menstrual cycle, with a tendency to peak in the preovulatory phase (though non-significant compared to any other cycle phase). Interestingly, there was no correlation between PGRMC1 levels and estradiol or progesterone variations. We next analyzed PGRMC1 levels in blood samples obtained from 6 patients with polycystic ovary syndrome (PCOS) and 8 patients with premature ovarian failure (POF). In addition, we included four healthy men and five healthy postmenopausal women as reference groups. The results from the PGRMC1 analyses in PCOS and POF patients, postmenopausal subjects and healthy males were compared to the PGRMC1 levels during the early follicular phase (eFP) of healthy women with ovulatory cycles in order to obtain comparable endocrine conditions. We observed a marked reduction in levels of PGRMC1 protein in the PCOS and POF groups in comparison to healthy cycling women in the early follicular phase (figure 2B). Furthermore, a similar reduction was observed among postmenopausal women as well as in healthy males when compared to healthy females in eFP (*p*-values < 0.01; figure 2B). The lowest PGRMC1 levels were observed in the group of postmenopausal women. The levels were significantly reduced when compared to healthy females and also when compared to healthy males (*p*-values < 0.05; figure 2B). POF patients displayed a broader range in PGRMC1 expression than PCOS patients, while PGRMC1 levels in PCOS patients fell between levels observed in postmenopausal controls and in males.

## Discussion

Progesterone receptor membrane component 1 (PGRMC1) is a membrane progesterone-binding protein implicated in female reproduction [1,3,5,7,9,10,13-15]. PGRMC1 is widely expressed also in non-reproductive tissue and we have for the first time established PGRMC1 expression profiles in PNBC across the human menstrual cycle. PGRMC1 expression is unchanged across the menstrual cycle (as has been observed previously [11]) and does not correlate with variations in progesterone levels, suggesting that PGRMC1 and progesterone are not directly regulating each other in PNBC. This observation is in contrast to previous findings indicating that PGRMC1 is negatively regulated by progesterone and estrogen [16,17]. Our observation may reflect different responses to progesterone in distinct cell types.

We investigated expression levels of PGRMC1 in patients with reduced fertility, i.e. premature ovarian failure (POF) and polycystic ovary syndrome (PCOS). The conditions were classified as idiopathic in all patients and none of the patients had regular menses. Interestingly, PGRMC1 is significantly down-regulated in patients from both the POF and the PCOS groups, supporting a connection between PGRMC1 levels in PNBC and ovarian function [11].

Our results indicate that PGRMC1 levels in PNBC are reduced in patients with impaired or absent ovulatory function. PGRMC1 binds and activates cytochrome P450 proteins involved in the metabolism of drugs, hormones, lipids and the synthesis of sterols. PGRMC1's function in leukocytes is still unclear. We could not detect the nuclear progesterone receptor in samples from PNBC by RT/PCR (unpublished data) and we hypothesize that PGRMC1 could be a candidate in mediating progesterone's effects on inflammation, coagulation and platelet function [18-20]. In higher organisms PGRMC1 has additional binding partners, e.g. progesterone binding protein (Prog BP) and plasminogen activator inhibitor RNA-binding protein (PAIRBP1) of importance for cell survival and apoptosis [6]. Most of these functions are critical for any tissue and may be specified by the interacting downstream molecules that are expressed. The induction of metabolic pathways at the cellular level mediated by PGRMC1 is likely to be tissue specific. Despite yet unknown downstream partners, our finding of an association between PGRMC1 levels in PNBC and ovulatory function may suggest an important effect for the general reproductive fitness. It may be hypothesized that in the absence of ovulation a systemic induction by PGRMC1 is not initiated.

In summary, this study demonstrates that PGRMC1 levels in PNBC are strongly associated with ovulatory function. The levels in POF and PCOS patients are significantly reduced when compared to healthy menstruating females. The latter group shows large inter- and intraindividual variations in PGRMC1 levels with a tendency for a peak at the preovulatory phase. Further studies on PGRMC1 expression following larger patient cohorts over time are warranted.

## Competing interests

The authors declare that they have no competing interests.

## Authors' contributions

JS and TK performed experiments and analyzed data. POK and ISP examined and recruited patients and control subjects. ND supervised the study and analyzed data. JS, ISP and ND performed statistical analysis and interpretation of data. JS wrote the manuscript with help of ISP and ND. All authors read and approved the final manuscript.
